# Clinical Application of Pharmacokinetics to Appraise Adherence to Levetiracetam in Portuguese Epileptic Patients

**DOI:** 10.3390/biomedicines10092127

**Published:** 2022-08-30

**Authors:** Rui Silva, Joana Bicker, Anabela Almeida, Andreia Carona, Ana Silva, Francisco Sales, Isabel Santana, Amílcar Falcão, Ana Fortuna

**Affiliations:** 1 Laboratory of Pharmacology, Pólo das Ciências da Saúde, Azinhaga de Santa Comba, Faculty of Pharmacy, University of Coimbra, 3000-548 Coimbra, Portugal; 2 CIBIT/ICNAS—Coimbra Institute for Biomedical Imaging and Translational Research, University of Coimbra, 3000-548 Coimbra, Portugal; 3 CIVG—Vasco da Gama Research Center/EUVG—Vasco da Gama University School, 3020-210 Coimbra, Portugal; 4 Refractory Epilepsy Reference Centre, Centro Hospitalar e Universitário de Coimbra, EPE, 3004-561 Coimbra, Portugal

**Keywords:** adherence, epilepsy, pharmacokinetic monitoring, levetiracetam

## Abstract

Adherence to antiseizure drug treatment determines its effectiveness and safety, and consequently affects patients’ quality of life. Herein, we assessed adherence to levetiracetam in Portuguese patients with refractory epilepsy (*n* = 115), with resort to a pharmacokinetic drug monitoring approach. The pharmacokinetic parameters of levetiracetam in each patient were determined in steady-state while admitted to the hospital. Then, adherence was assessed by comparing the plasma concentration of the drug observed on the first day of hospitalization with the predicted plasma concentration, considering previously determined pharmacokinetic parameters. The rate of adherence was assessed according to gender, age, diagnosis, and antiseizure drug regimen. Among 115 enrolled patients, 49 (42.6%) were identified as non-adherent, 30 (26.1%) classified as under-consumers, and 19 (16.5%) as over-consumers. A relationship between adherence, daily dose and plasma concentrations was herein reported for the first time. Adherent patients received higher daily doses of levetiracetam [2500 (2000–3000) mg] than non-adherent over-consumers [1500 (1000–2000) mg] and non-adherent under-consumers [2000 (1500–3000) mg]. Higher average steady-state plasma concentrations of levetiracetam were found in non-adherent under-consumers [27.28 (15.33–36.36) mg/L], followed by adherent patients [22.05 (16.62–29.81) mg/L] and non-adherent over-consumers [17.50 (10.69–24.37) mg/L]. This study demonstrates that adherence (or lack thereof) influences the plasma concentrations of levetiracetam in steady-state and its pharmacological effects. Moreover, it emphasizes the importance of educating patients to encourage adherence to therapy. Otherwise, the risk of developing toxic and subtherapeutic concentrations is undeniable, compromising the therapeutic effect and safety of treatment.

## 1. Introduction

Adherence to antiseizure drug treatment hugely influences drug effectiveness, disease progression and, consequently, the quality of life of epileptic patients. Indeed, poor adherence to antiseizure medication is associated with increased mortality, morbidity and healthcare costs [1,2,3,4]. Uncontrolled epilepsies due to inadequate adherence can lead to serious clinical events such as falls, fractures and head injury, which often require hospitalization. Therefore, in addition to negative clinical outcomes, nonadherence is associated with an increase of health care utilization and medical costs. Furthermore, uncontrolled epilepsies can result in social disabilities such as reduced social interactions, difficulty in finding a job or loss of driving license [5]. Adherence to antiseizure drug treatment has been reported as poor among epileptic patients, with nonadherence rates ranging between 26% [5] and 79% [6], depending on the study design [2].

Adherence is defined as compliance to the drug therapeutic regimen that was prescribed by the clinician and agreed on by the patient [1]. Therefore, nonadherence comprises any deviation from a prescribed drug therapeutic regimen, including dose changes, frequency changes, overuse and underuse [1,2,7]. Multimodal causes are responsible for poor adherence and have been attributed to patients and their relationship with the clinician or health care systems. Patient-related causes include demographic, socioeconomic, disease-related and therapeutic-related factors [1,2,7].

It has been demonstrated that adherence to antiseizure drug treatment is lower in younger adults and male patients with generalized epilepsy [2]. Co-morbidities, such as depression or behavioral disorders, as well as lower socioeconomic status and educational attainment are also associated with poor adherence [1,2,8]. Likewise, chronicity and polytherapy are predictors of poor adherence to treatment. Polytherapy dosing schedules are less comfortable than monotherapy and, hence, more associated with the occurrence of side effects [2]. The tolerability and comfort of the prescribed dosing schedules are equally determinant factors for good adherence [2,8]. 

An early assessment of adherence to antiseizure therapy is crucial to identify nonadherent patients and the underlying causes of lack of compliance. In turn, this will avoid subtherapeutic and/or adverse side effects [1,2,8], which is particularly relevant since nonadherence can be responsible for inadequate seizure control. Additionally, it will enable the exclusion of drug resistance. Indeed, one third of epileptic patients are refractory to antiseizure drug treatment, and nonadherence can lead to drug failure and wrong diagnosis of refractory epilepsy [8,9,10].

Adherence to treatment can be measured through direct or indirect methods. Indirect methods encompass patient self-reports, diaries and filled questionnaires, while direct methods comprise electronic medication monitoring, prescription refills, pill counts, directly observed therapy and measurement of drug levels in biological fluids [2,7]. Although each method has its own advantages and limitations, direct methods are, in general, more accurate but also more expensive and require experienced professionals. In turn, indirect methods are easier to perform and more affordable, but susceptible to distortion [7]. None of these methods are considered gold standard and the use of multiple approaches provides a more accurate assessment of adherence [2,7].

Measuring adherence through the evaluation of drug levels has been applied in epilepsy since the 1970s, when therapeutic drug monitoring of antiseizure drugs became available worldwide [2,7]. However, it is known that single drug levels are a poor marker to predict adherence or nonadherence. Single trough levels should always be interpreted regarding the individual therapeutic concentration range of the patient, and considering their optimal adherence concentration range [11,12,13,14]. Thus, adherence evaluation must be carried out while resorting to established therapeutic drug monitoring protocols that ought to include multiple and specific sampling points, namely concentrations that reflect the patients usual adherence in ambulatory and concentrations obtained in optimal adherence conditions [13,14]. Moreover, the results should always be interpreted considering the clinical context of the patient, the pharmacokinetic variability of each drug and the accuracy of the analytical techniques [14,15].

Levetiracetam has been one of the most frequently administered antiseizure drugs in the last decade, mainly due to its efficacy and safety profiles on the treatment of patients with focal and generalized epilepsy [16,17,18,19]. This second-generation antiseizure drug acts through the modulation of excitatory neurotransmission by binding to synaptic vesicle glycoprotein 2A. Furthermore, levetiracetam has been ascribed as the antiseizure drug with nearly ideal pharmacokinetic properties, namely a quick and complete absorption, negligible protein binding, absence of hepatic metabolism and major renal excretion [20,21]. Despite its favorable characteristics, therapeutic monitoring of levetiracetam has been reported as useful in critically ill patients, including refractory patients, paediatric and elderly patients and during pregnancy [22,23,24,25,26].

Since adherence to antiseizure drug treatment is an unquestionable issue and levetiracetam is one of the most frequently used antiseizure drugs, we were prompted to investigate the adherence of refractory epileptic patients to levetiracetam therapy through a pharmacokinetic monitoring approach.

## 2. Patients and Study Design

A retrospective observational study was herein performed enrolling 115 patients admitted to the Refractory Epilepsy Centre of the Centro Hospitalar e Universitário de Coimbra, EPE (CHUC, EPE, Coimbra, Portugal) for video-electroencephalography (VEEG) monitoring, between January 2018 and December 2021. VEEG monitoring is a diagnosis technique performed to clarify the type of epilepsy and epileptic seizures, as well as the location of the epileptic focus. All enrolled patients were under levetiracetam treatment for seizure control and submitted to therapeutic drug monitoring as part of their routine clinical management. The following data were collected for each patient: gender, age, diagnosis (type of epilepsy and location), and all details of the prescribed antiseizure drug regimen (drugs and posology). The glomerular filtration rate (eGFR, mL/min/1.73 m^2^) was estimated by the Modification of Diet in Renal Disease study method [27]. The drug load was calculated for each patient as the ratio between the prescribed daily dose (PDD) and the World Health Organization-defined daily dose (DDD) [28] of all the antiseizure drugs included in the individual drug regimen [29] (Table 1). Table S1 containing raw data can be observed in supplementary material.

All patients agreed to participate in the study and signed the informed consent. The study was approved by the Ethics Committee of the Faculty of Medicine of the University of Coimbra, Coimbra, Portugal (CE-061/2018) on 23 July 2018 and by the Ethics Committee of CHUC, EPE (CHUC-144-18) on 3 July 2019.

**Figure 1:**
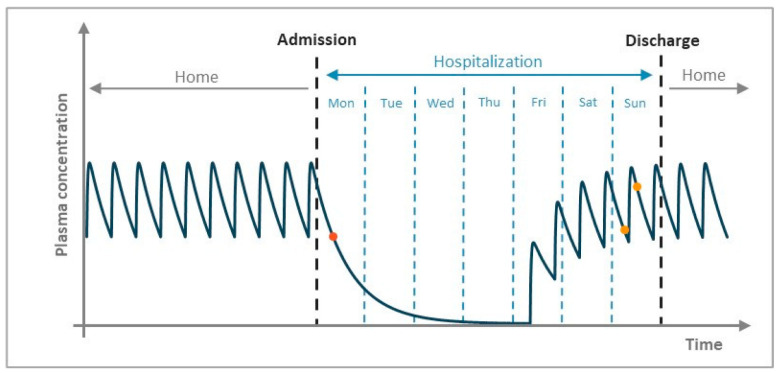
Schematic representation of a concentration/time profile of levetiracetam before, during and after hospitalization. The orange point represents the plasma concentration of levetiracetam obtained on the first day of hospitalization. The two yellow points represent plasma concentrations in steady-state obtained on the last day of hospitalization, which were used to estimate pharmacokinetic parameters.

The present study was conducted according to the daily routine of the Refractory Epilepsy Centre and the established therapeutic drug monitoring protocol. Patients are admitted to the hospital on a Monday, and their discharge occurs on the following Saturday or Sunday. In the morning of the admission day, patients do not take the prescribed antiseizure drugs because drug discontinuation is required to precipitate seizures for VEEG monitoring [30,31,32,33,34]. Following the reintroduction of antiseizure drugs (usually on Wednesday or Thursday), patients remain in the hospital for 2 to 3 additional days, to ensure that drug plasma concentrations reach therapeutic levels. Figure 1 depicts a typical concentration/time profile of levetiracetam before, during and after hospitalization of the epileptic patients included in the study, as well as the strategic points of blood collection.

### 2.1. Sampling Protocol, Drug Measurement and Pharmacokinetic Analysis

Briefly, the therapeutic drug monitoring protocol implemented in the Refractory Epilepsy Centre of CHUC, EPE, consists of collecting a blood sample before drug administration (30 min pre-dosing), in the morning of the first hospitalization day, and two blood samples (30 min pre-dosing and 1 h post-dosing) in the last day of hospitalization (Figure 1). Since drug intake during hospitalization is performed under nurse supervision, plasma concentrations obtained on the last day of hospitalization reflect the data under optimal adherence conditions. Thus, pharmacokinetics of levetiracetam were characterized using plasma concentrations collected during this period.

Blood samples were collected to heparin-lithium tubes and centrifuged to obtain plasma. The date and time of each sample collection were recorded. The determination of levetiracetam plasma concentrations was performed by liquid–liquid extraction followed by high performance liquid chromatography with diode array detection, as described and validated by Gonçalves et al. [35].

The apparent volume of distribution (Vd/F) expressed in L/kg and oral clearance (CL/F) expressed in L/h/kg were estimated through compartmental analysis, resorting to the nonlinear least squares method of the Abbottbase Pharmacokinetic System software (PKS^®^ System, version 1.10, Abbott Diagnostics, Green Oaks, IL, USA). The corresponding elimination half-life (t_1/2_), expressed in h, and average steady-state plasma concentration (C_AV,SS_), expressed as mg/L, were estimated. The absorption constant rate (ka) was fixed at 2.44 h^−1^ in accordance with Rhee et al. [36]. A one-compartment model with first-order absorption and elimination was chosen because it has been reported as the most accurate model to describe the pharmacokinetics of levetiracetam [36,37,38,39].

### 2.2. Assessment of Patient Adherence to Levetiracetam

The plasma concentration of the first day of hospitalization was predicted according to individual levetiracetam pharmacokinetic parameters, calculated resorting to plasma concentrations obtained under optimal adherence conditions. Then, the plasma concentration determined by HPLC on the admission day (which reflects the plasma concentration at home) was compared with the predicted plasma concentration (which reflects the plasma concentration under optimal adherence). The percentage of deviation between both concentrations (Equation (1)) is calculated to identify adherent and non-adherent patients:

(1) $$\%Deviation=\left(\frac{Observed\;plasma\;concentration-Predicted\;plasma\;concentration}{Predicted\;plasma\;concentration}\right)\times 100$$

Patients were considered adherent to levetiracetam treatment if the absolute difference between observed and predicted plasma concentrations was ≤30%. Non-adherent patients were considered under-consumers if the difference was ≤−30%, and over-consumers if the difference was ≥+30%. The threshold of 30% was herein adopted in accordance with the adherence study reported in [40] and based on [41]. Accordingly, the fluctuation of the plasma concentrations of the analyzed antiseizure drugs, under constant dosages, was always lower than 30%. Thus, patients presenting deviations higher than 30% can be safely classified as non-adherent.

### 2.3. Data Analysis

Adherence was evaluated according to gender (males/females), age, type (focal/generalized) and location of epilepsy (frontal/temporal/posterior), levetiracetam therapy (daily dose and average steady-state plasma concentration) and whole antiseizure drug therapy (number of antiseizure drugs and drug load). 

Statistical analysis was performed using Statistical Package for the Social Sciences 26.0 (IBM SPSS^®^, Armonk, NY, USA). The Shapiro–Wilk test was used to test normality. Since a deviation from normal distribution was found for all variables, the results were expressed as median and 25th and 75th quartiles, and the comparison between groups was assessed through the Mann–Whitney test. The results of the qualitative variables were expressed as absolute and relative frequencies. The Pearson Chi-square test was performed to compare frequencies. Statistical significance was set at *p* ≤ 0.05.

## 3. Results

A total of 115 patients were included in the pharmacokinetic study and screened for adherence to levetiracetam therapy. The observed results of the pharmacokinetic parameters are summarized in Table 2.

Regarding adherence, 66 (57.4%) patients presented absolute differences between observed and predicted plasma concentrations ≤30%, and were, consequently, classified as adherent patients to levetiracetam therapy. On the other hand, 49 (42.6%) presented absolute differences >30% and were considered non-adherent. Among these, 30 (61.2%) were classified as under-consumers (differences ≤ −30%) and 19 (38.8%) as over-consumers (differences ≥ +30%). Table S2 summarizes the characteristics of each patient group defined according to patient adherence.

**Figure 2:**
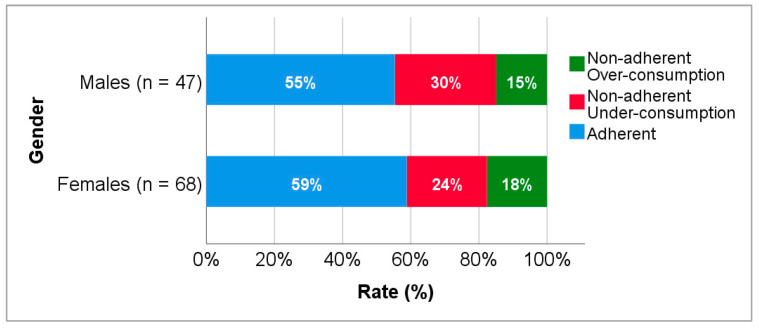
Adherence to levetiracetam treatment according to gender.

Subpopulations were established to assess the impact of patient, disease and drug-related factors that may influence adherence. Male and female patients showed similar rates of adherence (55.3% vs. 58.8%), as demonstrated in Figure 2. Among non-adherent male patients, 14 (29.8%) were under-consumers and 7 (14.9%) were over-consumers, while 16 (23.5%) of non-adherent female patients were under-consumers and 12 (17.6%) were over-consumers. No statistically significant differences were found between the groups.

The median age observed in adherent patients [33.50 (24.00–45.25) years] was lower than the values observed in non-adherent under-consumers [42.00 (24.00–46.25) years] and non-adherent over-consumers [48.00 (31.00–54.00) years], but not statistically significant.

**Figure 3:**
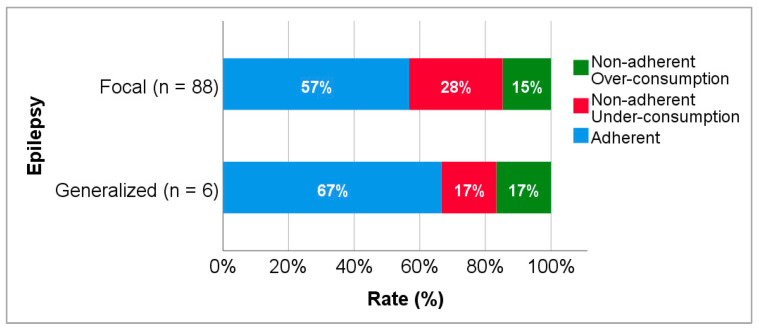
Adherence to levetiracetam treatment according to type of epilepsy.

Amongst the patients enrolled in the study, 88 (76.5%) were diagnosed with focal epilepsy and only 6 (5.2%) with generalized epilepsy. Concerning the patients with focal epilepsy, 50 (56.8%) were adherent and 38 (43.2%) were non-adherent. With regard to the six patients with generalized epilepsy, four (66.7%) were adherent and two (33.3%) non-adherent (Figure 3).

**Figure 4:**
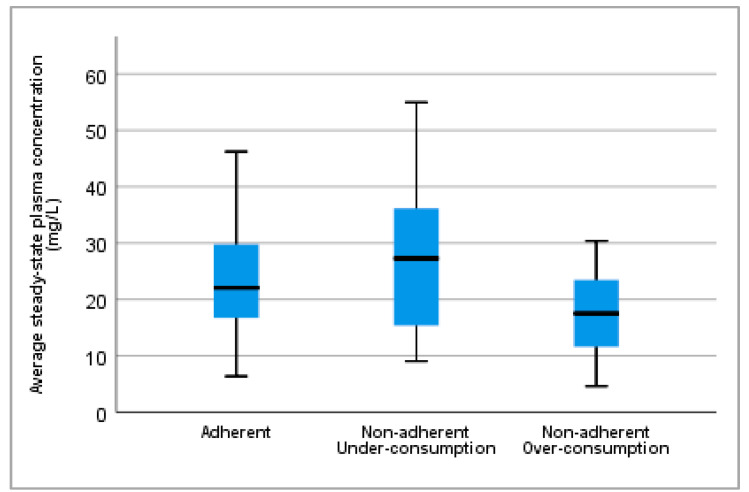
Average steady-state plasma concentration of levetiracetam in each group of patients previously classified according to adherence. Statistically significant differences (*p* = 0.041) were observed between non-adherent under-consumers and non-adherent over-consumers. Results are expressed as median and 25th and 75th quartiles. Black lines represent the median and blue blocks the 25th and 75th quartiles. Error bars represent 1.5 times the interquartile range from the 25th and 75th quartiles.

Figure 4 depicts the average steady-state plasma concentrations of levetiracetam for each patient group, classified in accordance with the patient’s adherence. Adherent patients received higher median daily doses of levetiracetam [2500 (2000–3000) mg] that significantly differed (*p* = 0.030) from non-adherent over-consumers [1500 (1000–2000) mg] but not (*p* = 1.000) from non-adherent under-consumers [2000 (1500–3000) mg].

Adherent patients showed a median average steady-state plasma concentration value [22.05 (16.62–29.81) mg/L] between non-adherent over-consumers [17.50 (10.69–24.37) mg/L] and non-adherent under-consumers [27.28 (15.33–36.36) mg/L]. Statistically significant differences (*p* = 0.041) were found between the median average steady-state plasma concentration of non-adherent under-consumers and non-adherent over-consumers, but not between the other groups. Table S3 displays the absolute and relative frequencies of patients in accordance with their daily dose and adherence classification.

**Figure 5:**
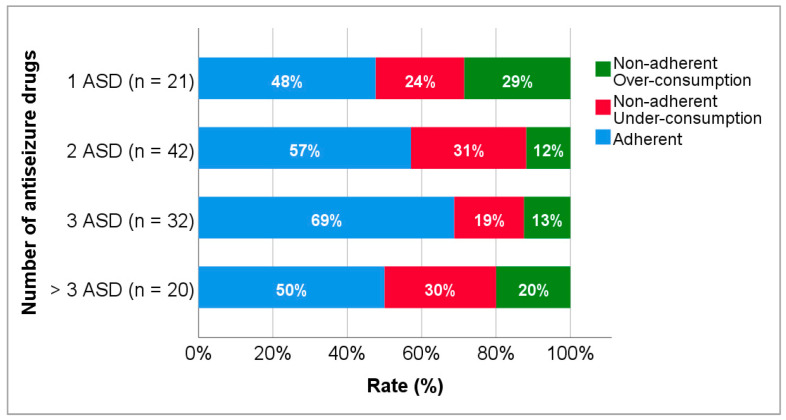
Adherence to levetiracetam treatment according to the number of antiseizure drugs. ASD, antiseizure drug.

**Figure 6:**
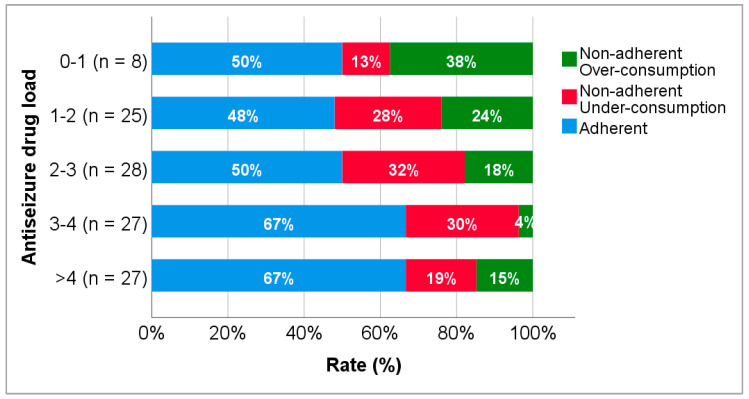
Adherence to levetiracetam according to the antiseizure drug load.

Adherence rates according to the number of antiseizure drugs and antiseizure drug load can be observed in Figure 5 and Figure 6, respectively. The rate of adherence in patients on monotherapy was 47.6% but increased to 57.1% and 68.8%, in patients on two and three antiseizure drugs, respectively, and decreased in patients on four or more antiseizure drugs (50.0%). A higher rate of over-consumption (28.6%) was observed in patients on monotherapy. No statistically significant differences were found between groups. Regarding adherence according to drug load, the rate of adherence was similar (≈50%) in patients with drug loads under 3, but higher in patients with drug loads higher than this value. A superior rate of over-consumption (37.5%) was found among patients with drug loads below 1. No statistically significant differences were observed.

## 4. Discussion

In the present study, a clinical pharmacokinetic approach to assess adherence to treatment with antiseizure drugs was proposed. Previous studies applied plasma concentrations of antiseizure drugs with the purpose of assessing adherence to antiseizure drug treatment [6,13,40], but none applied pharmacokinetic evaluations.

Herein, the suggested pharmacokinetic approach firstly enables the identification of the individual plasma concentrations of each patient on optimal adherence. Then, it accurately quantifies the deviation from optimal adherence, using plasma concentrations regardless of the time of collection or dosing changes.

This strategy was applied to patients submitted to VEEG monitoring and administered levetiracetam as part of their antiseizure therapy. The VEEG session is carried out to establish a complete diagnosis of the epileptic condition of each patient, in parallel with therapeutic drug monitoring of antiseizure drugs to adjust dosing regimens. The VEEG exam comprises a drug discontinuation protocol as a strategy to precipitate seizures, identically to other epilepsy diagnostic centers worldwide [13,30,32,33,34]. In the case of polytherapy, levetiracetam is usually the first drug to be withdrawn due to it short half-life, which allows a faster decrease of plasma concentrations after discontinuation [32,34] and a quick reinstatement of therapeutic levels after seizure occurrence, through intravenous administration.

A 42.6% rate of nonadherence to levetiracetam treatment was found in this study. This result is in agreement with other studies that evaluated adherence to antiseizure drug treatment through the direct measurement of drug plasma concentrations [6,40]. Carpentier et al. [40] analyzed 48 patients with refractory focal epilepsy and found an overall rate of adherence to antiseizure drugs of 40.9%. Similarly, Samsonsen et al. [6] assessed 282 epileptic patients acutely hospitalized for seizures and found a rate of nonadherence of 39.0%. These authors also mentioned that, among the analyzed antiseizure drugs, levetiracetam showed the highest rates of nonadherence, probably due to its shorter plasma half-life. The high rate of nonadherence may also be related with the high number (73%) of patients diagnosed with focal epilepsy with temporal lobe involvement included in the present study. Patients with temporal lobe epilepsy are more associated with long-term memory impairment that can contribute to an irregular intake of the antiseizure drugs, as previously suggested by Carpentier et al. [40].

Complementarily, adherence to levetiracetam treatment was also assessed through the widely adopted direct method of medication possession ratio, defined as the ratio of total days supplied for a specific drug within an observation period to total days in the observation period [3,4]. Accordingly, levetiracetam was the antiseizure drug with the highest rate of adherence. Ettinger et al. [4] reported that levetiracetam has the highest mean medication possession ratio (87%), comparable with the results of Davis et al. [3] (82%), which observed a rate of adherence to levetiracetam (67.9%) above the observed overall rate of 60.7%.

The present study also found a rate of 26.1% of non-adherent under-consumers and 16.5% non-adherent over-consumers. This is not in accordance with the lower rate of under-consumers (9.1%) and the higher rate of over-consumers (27.3%) observed by Carpentier et al. [40]. The authors stated that over-consumption of antiseizure drugs is probably under-estimated in daily practice. The causes of nonadherence by under-consumption may include intentional and non-intentional situations such as forgetfulness or difficulties in understanding the prescribed dosing regimen. Intentional situations include the absence of seizures, lack of awareness of the need for treatment, an occurrence or persistence of an adverse effect, and lack of belief in treatment or comorbid clinical situations [8]. Indeed, epilepsy is a condition associated with a high rate of comorbidities such as migraine or psychiatric disorders. About 50% of adult patients with epilepsy have at least one comorbidity. Many of these disorders can affect patient behavior and consequently adherence to treatment [42]. In turn, lack of treatment can lead to seizure occurrence and to a reduction of the quality of life [8]. Furthermore, under-consumption of antiseizure drugs may be concealing situations of drug resistance [9,10].

On the other hand, nonadherence by over-consumption may be non-intentional due to a misunderstanding of the prescribed dosing regimen, or intentional, related to the fear of suffering from a seizure or so-called “white-coat” adherence, preceding the hospitalization to mask situations of poor adherence [8]. The over-consumption of antiseizure drugs can lead to concentration-dependent adverse events [11].

The effect of gender and age on adherence to levetiracetam was herein assessed. Male and female patients showed similar rates of adherence (55.3% vs. 58.8%), corroborating literature data [8,40]. Despite being lower, no statistically significant differences were found between the median age of adherent patients (33.50 years), and the median age of non-adherent under-consumers (42.00 years) and non-adherent over-consumers (48.00 years). These results are in accordance with the observations of Carpentier et al. [40]. It is worth noting that the present study comprises a population of patients with a narrow age range (25 to 40 years old), in opposition to Samsonsen et al. [6] who found significantly lower rates of adherence among the younger patients of a study population ranging from 16 to 91 years of age.

Drug dosing and plasma concentrations also affect adherence to antiseizure drugs [2,6]. However, the relationship between the administered dose and achieved plasma concentration may not be linear, due to pharmacokinetic variability [11]. Regarding levetiracetam, it has been demonstrated that its pharmacokinetics is influenced by factors such as gender [43,44], age [44,45,46,47,48,49,50], weight [44,51,52], renal function [36,39,44,53], daily dose [37,51,53], and concomitant drug therapy [38,39,43,45,50,54,55]. The oral clearance of levetiracetam decreases with age [44,45,47,48,49,50], especially in older patients [46], and also with patient weight [44,51,52]. Thus, older and overweight patients tend to present higher plasma concentrations. Likewise, patients with renal impairment also presented higher plasma concentrations, since its clearance increases with higher creatinine clearance [36,39,44,53]. Contrarily, patients administered with higher daily doses of levetiracetam [37,51,53] and concomitant enzyme-inducing antiseizure drugs that enhance oral clearance [38,39,43,45,50,51,55] presented lower plasma concentrations.

Herein, plasma drug concentrations were tested, in addition to daily doses. Adherent patients received a higher daily dose of levetiracetam (2500 mg) than non-adherent under consumers (2000 mg) and non-adherent over-consumers (1500 mg). The difference between adherent and non-adherent over-consumers was statistically significant (*p* = 0.030).

Although non-adherent, under-consumers received lower daily doses of levetiracetam than adherent patients, and higher median average steady-state plasma concentrations were observed (27.28 mg/L vs. 22.05 mg/L). This can be explained by the aforementioned pharmacokinetic variability and by the enhanced risk of developing concentration-dependent adverse events with higher doses, contributing to under-consumption. Nevertheless, adverse effects have not been recorded to support this hypothesis. It is important to mention that non-adherent over-consumers show the lowest average steady-state plasma concentrations (17.50 mg/L), with statistically significant differences (*p* = 0.041) compared with non-adherent under-consumers (27.28 mg/L). Lower plasma concentrations can be related with a lack of effectiveness that may contribute to nonadherence by over-consumption.

Adherence to antiseizure drug treatment can also be affected by treatment complexity. Patients on polytherapy are commonly associated with lower rates of adherence than patients on monotherapy. In addition, multidrug treatments are more likely to cause adverse events that are frequently the reason of nonadherence [1,2]. Thus, in the present study, the relationship between the antiseizure therapy and adherence was investigated. The rate of adherence found in patients on monotherapy with levetiracetam was 47.6%. Contrary to expectations, the rate of adherence increases in patients with two and three antiseizure drugs to 57.1% and 68.8% (Figure 5), respectively. However, Buck et al. [56], Faught et al. [5], and Chapman [57] also found that epileptic patients on polytherapy are more likely to adhere to antiseizure drugs than patients on monotherapy. Patients medicated with several antiseizure drugs experience seizures less frequently than those on monotherapy, and consequently become aware of how important it is to comply to the therapeutic regime [8].

The antiseizure drug load corresponds to the sum of the daily dose normalized by the daily dose of each antiseizure drug included in the prescribed therapeutic regimen. Hence, drug load is a measure of the totality of the antiseizure therapy, which embodies not only the number of antiseizure drugs, but also the value of the prescribed daily dose [29,58,59]. Although the number of antiseizure drugs is related to the drug load, the occurrence of adverse effects is more related to the drug load than to the number of antiseizure drugs [29]. For this reason, the effect of antiseizure drug load was also explored. The rates of adherence and nonadherence were similar in patients with a drug load under 3.0 (Figure 6). In contrast, patients with drug loads above 3.0 showed higher rates of adherence, following the same trend of the adherence results according to the number of antiseizure drugs. Regarding drug load and adverse events, Canevini et al. [59] admitted that the presence of newer drugs in antiseizure therapies, which exhibit more favourable tolerability profiles, may contribute to the poor relationship between drug load and the occurrence of adverse events. Likewise, the irregular relationship herein found between the number of antiseizure drugs (or drug load) with drug adherence can be explained by the same reasons.

Concerning non-adherent patients, higher rates of over-consumption were observed in the monotherapy group (Figure 5) and in the group with lower drug load (Figure 6). This finding may be explained by the fear of having a seizure due to a feeling of lack of protection, contrary to patients on polytherapy, who feel more protected [5,8,56,57].

## 5. Conclusions

The present study demonstrated that adherence to levetiracetam treatment by Portuguese patients with refractory epilepsy is poor. Nonadherence was identified in 42.6% of the patients, requiring the development of accurate methodologies that assess adherence, the underlying causes of nonadherence and apply innovative strategies to improve it. The herein used pharmacokinetic approach showed to be a reliable, accurate and useful tool to assess adherence in epileptic patients, since it is based on drug plasma concentrations observed under optimal adherence conditions, and it can be applied to several antiseizure drugs.

## Figures and Tables

**Table 1:** Summary of the demographic and clinical characteristics of the population under investigation.

Patients, *n*	115
**Gender, *n* (%)**	
Male	47 (40.9%)
Female	68 (59.1%)
**Age**, years	38.00 (25.00–47.00)
**eGFR (mL/min/1.73 m^2^)**	108.65 ± 18.89
**Diagnosis, *n* (%)**	
Focal epilepsy	88 (76.5%)
Generalized epilepsy	6 (5.2%)
Unknown or not established	21 (18.3%)
**Localization, *n* (%)**	
Frontal	7 (8.0%)
Frontal-temporal	6 (6.8%)
Temporal	53 (60.1%)
Temporal-occipital	5 (5.7%)
Occipital	5 (5.7%)
Multi-focal	2 (2.3%)
Unknown or not established	10 (11.4%)
**Daily dose of levetiracetam**, mg	2000 (1500–3000)
**Frequency, *n* (%)**	
QD	2 (1.7%)
BID	96 (83.5%)
TID	17 (14.8%)
**Antiseizure drugs per patient, *n* (%)**	
1	21 (18.3%)
2	42 (36.5%)
3	32 (27.8%)
4	16 (13.9%)
5	4 (3.5%)
**Antiseizure drug load**	2.83 (1.67–3.85)
**Concomitant antiseizure drugs, *n***	
Carbamazepine	24
Clobazam	12
Clonazepam	16
Eslicarbazepine acetate	22
Lacosamide	9
Lamotrigine	8
Oxcarbazepine	4
Perampanel	23
Phenobarbital	3
Phenytoin	2
Topiramate	8
Valproic acid	24
Zonisamide	10

eGFR, estimated glomerular filtration rate; QD, *quaque die* (once a day); BID, *bis in die* (twice a day); TID, *ter in die* (three time a day). Results are expressed as absolute and relative frequencies, median and 25th and 75th quartiles or mean ± standard deviation.

**Table 2:** Pharmacokinetic parameters of levetiracetam observed in the study population.

Pharmacokinetic Parameters	Vd/F (L/kg)	CL/F (L/h/kg)	t_1/2_ (h)	C_AV,SS_ (mg/L)
Median	0.607	0.059	7.44	21.45
25th–75th quartiles	0.508–0.783	0.048–0.073	6.13–8.69	15.29–29.83
Min-Max	0.218–2.140	0.020–0.170	3.41–20.21	4.58–54.97

CL/F, Oral clearance; C_AV,SS_, Average steady-state plasma concentration; t_1/2_, Elimination half-life time; Vd/F, Apparent volume of distribution. Results are expressed as median, 25th and 75th quartiles and minimum (min) and maximum (max).

## Data Availability

Not applicable.

## Supplementary Materials

The following supporting information can be downloaded at https://www.mdpi.com/article/10.3390/biomedicines10092127/s1, Table S1: Demographic, clinical and therapeutic characteristics of the analysed patients; Table S2: Summary of the characteristics of each patient group according to their adherence classification; Table S3: Absolute and relative frequencies of patients according to the daily dose of levetiracetam and adherence classification.
