# Supplementary material for: Clinical Application of Pharmacokinetics to Appraise Adherence to Levetiracetam in Portuguese Epileptic Patients
Source: Biomedicines. 2022 Aug 30;10(9):2127. doi: 10.3390/biomedicines10092127 (PMC9495958; doi:10.3390/biomedicines10092127)
Supplement: Supplementary file 1 [file biomedicines-10-02127-s001.zip › biomedicines-1834416-supplementary.pdf]

Table S1 – Demographic, clinical and therapeutic characteristics of the analysed patients

Patient	Gender (M/F)	Age (years)	eGFR (ml/min/1.73m^2)	Diagnosis	Location	Daily dose (mg)	Frequency	Number of antiseizure drugs	Drug load	$C_{AV,SS}$ (mg/L)	Deviation between observed and predicted plasma concentrations (%)	Adherence
1	F	57	81.26	Focal	Multi-focal	2000	BID	3	3.53	21.31	-53.85	Non-adherent Under-consumer
2	F	34	94.55	Focal	Occipital	2000	BID	3	3.20	13.81	20.30	Adherent
3	M	47	133.50	Focal	Temporal	2500	BID	3	4.07	11.14	-9.22	Adherent
4	F	21	91.63	Focal	Temporal	2500	BID	2	2.47	14.63	-8.45	Adherent
5	M	24	125.01	Focal	Temporal	3000	BID	4	7.33	28.66	-14.79	Adherent
6	F	40	137.19	Focal	Temporal	3000	BID	3	4.40	21.23	-22.26	Adherent
7	M	41	132.44	Focal	Temporal	3000	BID	4	6.00	27.84	-41.35	Non-adherent Under-consumer
8	F	56	85.31	Focal	Temporal	3000	BID	4	3.29	29.76	-40.68	Non-adherent Under-consumer
9	F	51	110.30	Focal	Temporal	3000	BID	3	3.00	28.30	56.95	Non-adherent Over-consumer
10	M	56	98.29	Focal	Temporal	3000	BID	5	5.05	17.22	-0.80	Adherent
11	M	43	123.01	Focal	Temporal	2500	BID	2	2.67	30.33	-87.51	Non-adherent Under-consumer
12	F	26	92.04	Focal	Temporal	2500	BID	3	5.00	38.03	-96.30	Non-adherent Under-consumer
13	F	28	125.12	Not defined	-	3000	TID	2	2.50	27.26	-11.65	Adherent
14	F	50	63.70	Not defined	-	1500	TID	1	1.00	24.37	90.75	Non-adherent Over-consumer
15	M	44	115.56	Not defined	-	3000	BID	2	3.60	16.39	-35.66	Non-adherent Under-consumer
16	F	19	110.26	Generalized	-	1500	BID	1	1.00	19.33	27.39	Adherent
17	F	22	133.31	Focal	Frontal	2000	BID	3	2.60	17.50	15.56	Adherent

18	M	25	113.40	Focal	Temporal	3000	BID	2	2.67	20.85	45.17	Non-adherent Over-consumer
19	F	22	123.40	Focal	Temporal	3000	TID	2	2.75	34.87	-37.52	Non-adherent Under-consumer
20	F	22	105.08	Focal	Temporal	2500	BID	2	3.17	30.90	-1.39	Adherent
21	F	45	111.38	Not defined	-	3000	BID	1	2.00	46.21	-8.22	Adherent
22	F	50	96.62	Focal	Frontal	2500	TID	2	3.00	26.72	-50.33	Non-adherent Under-consumer
23	M	29	119.88	Focal	-	2000	BID	3	3.33	17.68	0.12	Adherent
24	M	39	111.20	Focal	Temporal	2000	BID	2	2.67	17.77	8.03	Adherent
25	M	22	170.75	Focal	Temporal	1500	BID	2	2.00	20.85	32.30	Non-adherent Over-consumer
26	F	59	132.59	Focal	Temporal	2000	BID	2	3.25	40.88	-37.23	Non-adherent Under-consumer
27	M	26	97.38	Focal	Temporal-occipital	3000	BID	5	4.38	43.51	0.73	Adherent
28	F	40	108.60	Not defined	-	1000	BID	1	0.67	10.59	-18.19	Adherent
29	M	26	129.41	Focal	Temporal	1500	BID	2	1.50	21.14	-38.88	Non-adherent Under-consumer
30	M	71	118.48	Focal	Temporal	1500	BID	2	1.25	21.10	-13.83	Adherent
31	F	23	100.43	Focal	-	1500	BID	1	1.00	13.91	288.32	Non-adherent Over-consumer
32	F	24	88.11	Focal	Frontal	3000	BID	3	4.00	24.34	-13.02	Adherent
33	F	48	-	Focal	Temporal	3000	BID	2	4.00	10.69	103.39	Non-adherent Over-consumer
34	M	19	126.74	Focal	Occipital	3000	BID	2	3.50	16.78	17.01	Adherent
35	M	20	126.09	Focal	Occipital	3000	BID	3	3.75	22.10	22.83	Adherent
36	F	57	107.70	Focal	Temporal	4500	TID	3	4.08	37.18	-27.06	Adherent
37	F	42	94.56	Focal	Temporal	2000	BID	2	2.00	22.61	7.86	Adherent
38	F	28	113.89	Focal	Frontal	3000	TID	3	5.10	18.54	-13.34	Adherent
39	F	23	95.42	Not defined	-	2000	TID	3	3.25	22.16	-19.32	Adherent

40	F	43	94.25	Focal	Temporal	3000	BID	1	2.00	44.09	-54.93	Non-adherent Under-consumer
41	F	22	109.69	Generalized	-	1000	BID	2	1.81	9.77	-18.40	Adherent
42	M	39	111.20	Focal	Temporal	2000	BID	3	3.17	17.39	-51.09	Non-adherent Under-consumer
43	M	55	119.54	Focal	Temporal	1000	BID	2	1.67	9.01	-31.03	Non-adherent Under-consumer
44	F	40	106.57	Desconhecida	-	1000	QD	2	1.07	12.86	25.83	Adherent
45	F	19	112.37	Focal	Frontal-temporal	1500	BID	1	1.00	15.34	-41.59	Non-adherent Under-consumer
46	F	24	126.12	Generalized	-	3000	BID	2	4.00	39.84	-57.34	Non-adherent Under-consumer
47	M	26	103.72	Focal	Temporal	3000	TID	3	4.58	28.41	-19.28	Adherent
48	F	21	115.60	Focal	Frontal-temporal	3000	BID	4	4.20	18.42	21.72	Adherent
49	M	47	108.04	Focal	Temporal	3500	TID	4	4.75	30.36	84.28	Non-adherent Over-consumer
50	M	23	77.92	Focal	Temporal	3000	BID	2	3.00	22.51	-0.90	Adherent
51	M	44	120.86	Focal	-	3000	TID	3	4.80	29.48	-34.73	Non-adherent Under-consumer
52	M	20	126.74	Focal	Occipital	3000	BID	2	3.00	21.99	-24.85	Adherent
53	M	38	121.45	Focal	-	2000	BID	3	3.58	23.74	-21.22	Adherent
54	M	76	50.20	Not defined	-	500	BID	3	0.41	13.50	-8.33	Adherent
55	F	33	93.80	Focal	Temporal	3000	BID	3	4.83	38.05	8.18	Adherent
56	M	53	98.95	Focal	Temporal	1000	BID	4	4.02	6.32	44.66	Non-adherent Over-consumer
57	M	18	-	Focal	Temporal	2000	BID	3	2.13	11.85	-49.95	Non-adherent Under-consumer
58	F	30	-	Not defined	-	1000	BID	2	1.67	11.75	-0.10	Adherent
59	F	24	111.13	Not defined	-	2000	BID	1	1.33	33.06	-86.75	Non-adherent Under-consumer
60	F	59	96.32	Focal	Multi-focal	3000	TID	3	3.88	45.14	10.77	Adherent
61	F	27	121.71	Not defined	-	1500	BID	1	1.00	17.19	68.03	Non-adherent Over-consumer

62	F	46	87.25	Focal	Temporal	2250	BID	2	2.00	31.12	5.90	Adherent
63	F	33	117.02	Focal	Temporal	2000	BID	3	1.86	17.58	124.50	Non-adherent Over-consumer
64	M	22	104.03	Focal	Temporal-occipital	2500	BID	2	2.17	22.38	-11.77	Adherent
65	F	42	137.19	Focal	Temporal	3000	BID	3	5.07	25.38	0.14	Adherent
66	M	20	121.01	Not defined	-	1000	BID	1	0.67	10.81	-32.73	Non-adherent Under-consumer
67	M	27	-	Focal	-	3000	BID	5	6.50	19.03	-2.80	Adherent
68	M	23	77.92	Focal	-	3000	BID	2	3.00	27.92	-2.23	Adherent
69	F	41	117.85	Focal	Frontal-temporal	2000	BID	3	3.53	10.09	7.63	Adherent
70	M	43	96.43	Focal	Temporal	1500	BID	4	2.67	11.63	-68.53	Non-adherent Under-consumer
71	F	35	-	Focal	-	3000	BID	2	3.50	37.79	27.69	Adherent
72	F	61	-	Focal	Frontal	1000	BID	4	4.45	6.35	-0.54	Adherent
73	F	31	125.99	Focal	Temporal	3000	BID	2	3.20	22.12	-12.99	Adherent
74	F	43	116.95	Not defined	-	1000	BID	3	2.87	5.05	30.46	Non-adherent Over-consumer
75	F	22	125.46	Focal	Temporal	3500	BID	3	4.93	54.97	-47.02	Non-adherent Under-consumer
76	F	48	89.67	Focal	Temporal	2000	BID	2	2.67	26.96	55.00	Non-adherent Over-consumer
77	F	62	84.87	Focal	Frontal-temporal	2000	BID	2	2.13	30.49	-13.26	Adherent
78	F	47	91.97	Focal	Temporal-occipital	3000	TID	3	3.33	21.45	-8.22	Adherent
79	F	39	84.70	Focal	Temporal	1000	BID	2	2.00	9.86	9.62	Adherent
80	F	25	97.40	Not defined	-	1500	BID	2	1.42	17.70	10.61	Adherent
81	F	44	104.44	Focal	Temporal	2000	BID	2	1.73	17.99	-39.57	Non-adherent Under-consumer
82	M	25	136.37	Focal	Temporal	1000	BID	5	3.57	10.11	-36.18	Non-adherent Under-consumer
83	F	66	112.66	Focal	Temporal	1500	BID	2	1.50	31.30	-0.82	Adherent

84	F	54	89.43	Focal	Temporal	2000	BID	4	4.27	28.91	41.00	Non-adherent Over-consumer
85	F	19	122.67	Not defined	-	2000	BID	1	1.33	21.96	-21.95	Adherent
86	F	44	97.71	Focal	Frontal-temporal	4000	BID	4	3.85	36.94	-40.31	Non-adherent Under-consumer
87	M	28	129.41	Focal	Frontal-temporal	2000	BID	2	2.08	33.37	-20.89	Adherent
88	M	34	115.29	Focal	Temporal	2000	BID	1	1.33	17.50	33.21	Non-adherent Over-consumer
89	F	60	85.95	Focal	Temporal	2000	BID	1	1.33	36.17	-68.97	Non-adherent Under-consumer
90	F	46	102.57	Focal	Temporal	2000	BID	3	2.83	37.79	17.39	Adherent
91	M	48	107.47	Focal	Frontal	3000	TID	4	6.67	29.83	25.89	Adherent
92	F	38	107.66	Not defined	-	2500	TID	4	3.58	26.01	24.67	Adherent
93	F	26	127.16	Generalized	-	1000	BID	1	0.67	16.28	-4.36	Adherent
94	F	73	75.15	Focal	-	1000	BID	1	0.67	22.55	155.25	Non-adherent Over-consumer
95	F	63	73.89	Generalized	-	500	QD	4	1.21	12.49	98.78	Non-adherent Over-consumer
96	F	47	84.79	Focal	-	4000	BID	4	7.77	36.83	16.49	Adherent
97	M	44	107.37	Focal	-	3000	BID	1	2.00	33.46	16.76	Adherent
98	M	38	132.11	Focal	Temporal	2500	BID	3	3.33	24.20	-29.57	Adherent
99	M	31	146.55	Focal	Occipital	2000	BID	3	2.75	17.25	46.85	Non-adherent Over-consumer
100	M	31	132.88	Not defined	-	2000	BID	1	1.33	14.62	12.85	Adherent
101	M	20	-	Focal	Temporal	1500	BID	4	2.85	10.89	-55.87	Non-adherent Under-consumer
102	M	18	110.86	Focal	Temporal-occipital	2500	BID	3	4.67	31.72	-15.68	Adherent
103	M	36	121.61	Focal	Temporal-occipital	3000	TID	3	3.73	29.80	-13.59	Adherent
104	M	45	108.90	Focal	Temporal	2000	BID	2	2.67	17.81	-69.44	Non-adherent Under-consumer
105	F	75	98.64	Not defined	-	500	BID	2	0.67	4.58	166.12	Non-adherent Over-consumer

106	F	28	116.35	Focal	Frontal	1000	BID	1	0.67	10.68	-25.21	Adherent
107	F	32	83.19	Focal	Temporal	2000	BID	1	1.33	20.61	5.89	Adherent
108	F	52	102.92	Focal	Temporal	1500	BID	2	1.13	15.29	-63.14	Non-adherent Under-consumer
109	F	25	98.21	Generalized	-	3000	TID	4	4.25	16.73	8.88	Adherent
110	M	61	132.11	Focal	Temporal	1000	BID	2	1.87	6.58	17.86	Adherent
111	M	53	83.32	Focal	Temporal	1500	BID	1	1.00	12.22	2.99	Adherent
112	F	43	-	Not defined	-	2000	BID	2	2.33	27.25	20.95	Adherent
113	M	27	-	Focal	Temporal	3000	BID	2	2.80	38.31	-54.96	Non-adherent Under-consumer
114	F	38	114.75	Not defined	-	3000	TID	2	2.83	32.05	-68.80	Non-adherent Under-consumer
115	M	72	-	Not defined	-	500	BID	1	0.33	8.06	62.40	Non-adherent Over-consumer

$C_{AV,SS}$ – steady-state average plasma concentration; eGFR – estimated glomerular filtration rate;. F – female; M – male; QD, *quaque die* (once a day); BID, *bis in die* (twice a day); TID, *ter in die* (three time a day)

Table S2 – Summary of the characteristics of each patient group according to their adherence classification.

Group of patients/ Characteristic	Adherent	Non-adherent under-consumers	Non-adherent over-consumers
**Gender, n (%)**			
Male	26 (39.4%)	14 (46.7%)	7 (36.8%)
Female	40 (60.6%)	16 (63.3%)	12 (63.2%)
**Age, years**	33.50 (24.00 – 45.25)	42.00 (24.00 – 46.25)	48.00 (31.00 – 54.00)
**eGFR, mL/min/1.73 m^2**	108.15 ± 17.97	111.15 ± 15.78	106.46 ± 26.14
**Diagnosis, n (%)**			
Focal epilepsy	50 (75.8%)	25 (83.3%)	13 (76.5%)
Generalized epilepsy	4 (6.1%)	1 (3.3%)	1 (5.2%)
Unknown or not established	12 (18.2%)	4 (13.3%)	5 (18.3%)
**Localization, n (%)**			
Frontal	6 (9.1%)	1 (3.3%)	0 (0.0%)
Frontal-temporal	4 (6.1%)	2 (6.7%)	0 (0.0%)
Temporal	23 (34.8%)	20 (66.7%)	10 (52.6%)
Temporal-occipital	5 (7.6%)	0 (0.0%)	0 (0.0%)
Occipital	4 (6.1%)	0 (0.0%)	1 (5.3%)
Multi-focal	1 (1.5%)	1 (3.3%)	0 (0.0%)
Unknown or not established	23 (34.8%)	6 (20.0%)	8 (42.1%)
**Daily dose of levetiracetam, mg**	2500 (2000 – 3000)	1500 (1000 – 2000)	2000 (1500 – 3000)
**Frequency, n (%)**			
QD	1 (1.5%)	0 (0.0%)	1 (5.3%)
BID	54 (81.8%)	23 (86.7%)	16 (84.2%)
TID	11 (16.7%)	4 (13.3%)	2 (10.5%)
**CAV,SS, mg/L**	22.05 (16.62 – 29.81)	27.28 (15.33 – 36.36)	17.50 (10.69 – 24.37)
**Antiseizure drugs per patient, n (%)**			
1	10 (15.2%)	5 (16.7%)	6 (18.3%)
2	24 (36.4%)	13 (43.3%)	5 (36.5%)
3	22 (33.3%)	6 (20.0%)	4 (27.8%)
4	7 (10.6%)	5 (16.7%)	4 (27.4%)
5	3 (4.6%)	1 (3.3%%)	0 (0.00%)
**Antiseizure drug load**	3.09 (1.97 – 4.07)	2.82 (1.72 – 3.58)	2.00 (1.00 – 3.00)

QD, *quaque die* (once a day); BID, *bis in die* (twice a day); TID, *ter in die* (three time a day). Results are expressed as absolute and relative frequencies or median and 25th and 75th quartiles or mean ± standard deviation.

Table S3 – Absolute and relative frequencies of patients according to the daily dose of levetiracetam and adherence classification.

Daily dose	Adherent patients (n = 66)	Non-adherent under-consumers (n = 30)	Non-adherent over-consumers (n = 19)
**500 mg**	1 (25.0%)	0 (0.0%)	3 (75.0%)
**1000 mg**	9 (60.0%)	3 (20.0%)	3 (20.0%)
**1500 mg**	5 (35.7%)	5 (35.7%)	4 (28.6%)
**2000 mg**	15 (53.6%)	8 (28.6%)	5 (17.9%)
**2250 mg**	1 (100.0%)	0 (0.0%)	0 (0.0%)
**2500 mg**	7 (70.0%)	3 (30.0%)	0 (0.0%)
**3000 mg**	26 (68.4%)	9 (23.7%)	3 (7.9%)
**3500 mg**	0 (0.0%)	1 (50.0%)	1 (50.0%)
**4000 mg**	1 (50.0%)	1 (50.0%)	0 (0.0%)
**4500 mg**	1 (100.0%)	0 (0.0%)	0 (0.0%)
